# Lichens redefined as complex ecosystems

**DOI:** 10.1111/nph.16630

**Published:** 2020-06-02

**Authors:** David L. Hawksworth, Martin Grube

**Affiliations:** ^1^ Royal Botanic Gardens Kew Richmond Surrey TW9 3AE UK; ^2^ Natural History Museum Cromwell Road London SW7 5BD UK; ^3^ University of Southampton Southampton SO17 1BJ UK; ^4^ Institute of Biology University of Graz Holteigasse 6 8010 Graz Austria

**Keywords:** endolichenic fungi, galls, holobiont, lichen‐associated bacteria, lichenicolous fungi, mycobiont, photobiont, symbiosis, yeasts

## Abstract

This article is a Commentary on Mark *et al*. (2020), **227**: 1362–1375.

The work of Mark *et al*. (2020; pp. 1362–1375), in this issue of *New Phytologist*, prompted us to revisit how the term ‘lichen’ should now be defined, as they dig deeper into the biogeography and specificity of cystobasidiomycete yeasts and algae in lichens raising into question issues of specificity.

‘The symbiotic concept of “lichen” needs to take into account the diverse array of associated microscopic organisms.’

Lichens were the associations for which the term symbiosis (as ‘Symbiotismus’) was originally used in a biological sense by Albert Bernhard Frank (1876) following microscopic studies of five crustose lichens. From a lecture he gave in 1878, De Bary is often cited as the originator of the usage of the term for differently named organisms living together (Oulhen *et al.*, 2016), but he knew of Frank's work as he refers to it later (De Bary, 1884). The basic recognition of lichens as a dual association between a fungus and an alga, however, was made earlier by Schwendener in 1867 (Mitchell, 2007).

Irrespective of this new understanding of lichens as symbioses, they were already being recognized as hosts of other fungi in the preceding decades (Berkeley, 1844; Nylander, 1857), and Zopf (1897) referred to them as an additional part of the lichen symbiosis forming a ‘parasymbiosis’. Some formed necroses but others were symptomless, or were associated with brain‐like local outgrowths of certain lichen thalli; some 2319 species of mostly obligate lichenicolous (i.e. lichen‐inhabiting) fungi have now been named (Diederich *et al.*, 2018). In addition to those evidenced by spore‐bearing structures, it has long been known that many other fungi can be cultured from crushed lichen thalli (Petrini *et al.*, 1990); these asymptomatic fungi have been termed ‘endolichenic’. Sequencing shows that endolichenic fungi largely belong to families and genera also known as endophytes of plants (Tripathi & Joshi, 2019) rather than to the groups of obligate lichenicolous fungi; they can be very numerous, with up to 48 reported from a single lichen species (U'Ren *et al.*, 2012). The associated fungi in lichens may vary vastly in their biological impact to the association. Depending on abiotic and biotic conditions (such as the host lichen‐forming species) they may vary from being trapped resting spores, more or less unrecognized yeast or hyphal stages, to developers of distinct asexual and sexual structures. Although lichenicolous fungi may develop localized galls or hypertrophies on their hosts (e.g. De los Rios & Grube, 2001; Fig. 1), the architecture of the overall lichen structure in all cases remains determined by the principal mycobiont.

**Fig. 1:**
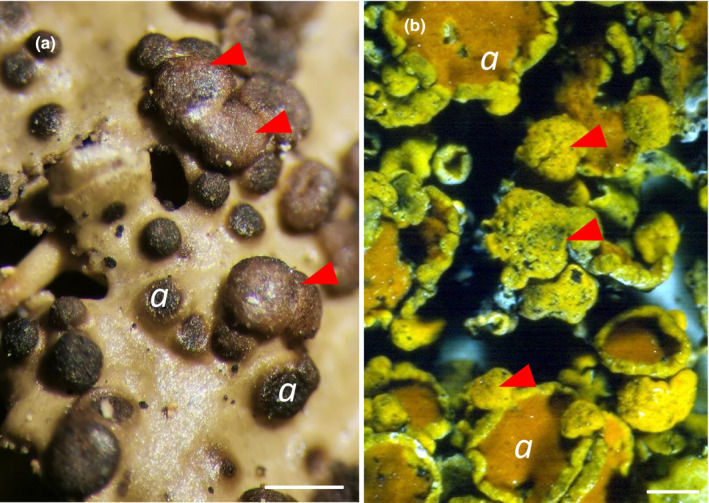
Gall‐forming lichenicolous fungi. (a) Convex galls (arrowheads) formed by the basidiomycete fungus *Tremella cetrariicola* on the lichenized *Tuckermannopsis chlorophylla* (photo: M. Grube). (b) Bullate galls (arrowheads) formed by the ascomycete fungus *Telogalla olivieri* on the lichenized *Xanthoria parietina*; the black spots on the galls are the openings of the spore‐producing structures of the *Telogalla* (photo: D. L. Hawksworth). The *Tremella* hypertrophises only the main fungal partner turning into a spore‐forming body, while the *Telogalla* hypertrophises the lichen structure and includes both the lichen‐forming fungus and its associated algae, and the yellow‐orange anthraquinone parietin continues to be produced. The letter *a* indicates the spore‐producing structures of the main lichen fungus. Bars, 1 mm.

Recently and controversially, the idea of a unitary role of the fungal partner in determining the characters of a lichen was challenged by an apparently hidden player in the lichen symbiosis. Basidiomycetous yeasts (*Cystobasidiomycetes*, *Pucciniomycotina*) were elegantly visualized by Spribille *et al*. (2016) for the first time in the thallus cortex of pendent lichens. High abundances of yeast cells were correlated with a yellow colour in the branches of one (*Bryoria fremontii*); individuals with different pigments in the branches had been classified as separate species before molecular work proving they were conspecific (Velmala *et al.*, 2009). Since Spribille *et al*. (2016) were able to detect representatives of this yeast lineage in a variety of lichens, using a PCR assay with highly specific primers, they concluded that these yeasts represented an integral component of the upper cortex of lichens. They further suggested a high degree of specificity, because each assayed lichen species carried a genetically distinct strain of the basidiomycete. These spectacular findings of a potential lichen ‘ménage à trois’, with two fungal partners suddenly exposed lichens to flashlights of scientific communication.

It was perhaps less noted that a new genus was described in the same year for two *Cystobasidiomycetes* yeasts on the basis of sexual stages developed on specific hosts, as typical for lichenicolous fungi (Millanes *et al.*, 2016). The significance of these lichen yeasts was discussed by the late Franz Oberwinkler (2017) who concluded that ‘it is obvious that basidiomycetous yeasts in lichen thalli are not a third component of symbiosis, but rather the vegetative propagules of mycoparasites’. Moreover, a broad survey for basidiomycete yeasts through metagenomic analysis of 339 lichen species (of 25 orders) confirmed the yeasts in only 2.7% of the sampled species (Lendemer *et al.*, 2019), although their metagenomic approach may be much less sensitive than PCR assays with specific primers. It remained, therefore, unclear, how ubiquitous and specific these yeast asexual stages actually were.

Mark *et al*. used specific primers to obtain sequences from the nuclear internal transcribed spacer (ITS) for fungal and algal partners as well as yeasts, sampling six widespread Northern Hemisphere epiphytic lichen species from 25 sites in Switzerland and Estonia. They demonstrated a frequent occurrence of cystobasidiomycete yeasts in the lichens. However, using interaction network and multivariate analyses, these yeasts were revealed as much less lichen‐specific than the included algae. Individuals of different lichen species from the same tree trunk consistently hosted the same or closely related lichen‐specific *Trebouxia* lineage over geographic distances, with the same algal strain sometimes being shared by different species. By contrast, the cystobasidiomycete yeasts were unevenly distributed over the study area and contrasting communities were found between Estonia and Switzerland. The results of Mark *et al*. suggest that cystobasidiomycete yeasts are not as intimately associated with the symbiosis as the algal partner. Further, their study did not reveal any specificity of cystobasidiomycete lineages at higher taxonomic ranks, as speculated by Černajová & Škaloud (2019). In the total fungal community associated with lichens (Fernández‐Mendoza *et al.*, 2017), fungi (including yeasts) seem to have a low specificity except for those known as lichenicolous fungi.

Similar questions about specificity have also been raised in relation to bacterial communities within lichens. While the deltaproteobacterium now known as *Melittangium lichenicola* was perhaps the first bacterium described from lichens (Thaxter, 1892), individual strains of bacteria have been isolated from lichens since the first half of the 20^th^ century. The dominance of *Alphaproteobacteria* as a particular group was first visualized by Cardinale *et al*. (2008) using fluorescence hybridization and confocal laser scanning microscopy. Subsequent studies of the diversity of lichen‐associated bacteria showed that the primary determinant of the composition is the fungal partner. Also, summarizing this work, Aschenbrenner *et al*. (2014) showed that for the lungwort lichen (*Lobaria pulmonaria*), there were shared (=core) and transient fractions of their bacterial biome, and that bacteria were included in the lichen's vegetative propagules (isidia), allowing a vertical transmission during asexual reproduction. Apparently, by the specific interaction with a suitable algal partner, the dominant lichen fungus is able to build up the basic structure (the thallus) for numerous associated and potentially interacting bacterial and other partners that colonize the lichen more or less specifically.

The resulting variation of the lichen symbiotic system is surprisingly manifold. We now know that the primary photobiont need not be restricted to a single strain of algae, but rather a co‐existence of multiple strains may contribute to the resilience of lichens (Casano *et al.*, 2011). Meanwhile protists, and even viruses, of lichens have been discovered in association with lichens (Wilkinson *et al.*, 2015; Petrzik *et al.*, 2019). As pointed out by Farrar (1976), lichens seem to have evolved as open systems, sometimes with special structures to facilitate gaseous exchanges, and can be interpreted as miniature ecosystems including a variety of organisms operating at different trophic levels.

The symbiotic concept of ‘lichen’ needs to take into account the diverse array of associated microscopic organisms. It could therefore be considered an example of a ‘holobiont’, with a dominant fungus and an included microbiome (Simon *et al.*, 2019); an evolved network of biotic associations that serve the fitness of the entire phenotype, with the morphology primarily shaped by the lichen fungus. As the main role of energy provision is played by photosynthetic partners, their variation is low and biomass is uniformly controlled, generally often under a fungal outer layer necessary for system maintenance. In comparison, the bacteria and yeasts associated with the outer layers are more diverse.

We can therefore re‐define the lichen symbiosis as: ‘A lichen is a self‐sustaining ecosystem formed by the interaction of an exhabitant fungus and an extracellular arrangement of one or more photosynthetic partners and an indeterminate number of other microscopic organisms’. The participants may grow separately under certain conditions in nature or in axenic cultures, and the resulting ‘lichen’ phenotype can be considered as the symbiotic phenotype of the lichen‐forming fungus (Honegger, 2012).

Finally, as it is sometimes a cause of confusion, we stress that the classification of lichen‐forming fungi is fully integrated into the system for fungi as a whole, considering formation of the lichen symbiosis as a fungal life‐style. All organisms within the symbiosis retain independent names, and the collective association itself has no separate name. This is especially pertinent for the at least 50 genera that include lichen‐forming fungi and also other species with different biologies; there are even cases where fungi are facultatively lichenized, forming associations with algae depending on ecological conditions. Understanding the physiological processes operating within complex lichen symbioses remains a scientific challenge, with much research still needed to elucidate the roles of the various component organisms, using comparative approaches (Grube *et al.*,  2015). Viewing lichens as self‐sustaining and adaptable systems of partnerships, however, overcomes the need to perpetuate debates as to particular unchanging numbers of essential symbionts.
